# Two Cases of Swallowing False Teeth

**Published:** 1886-10

**Authors:** 


### Two Cases of Swallowing False Teeth.

Billroth, in *Wiener Med. Blatter—Centralblatt fur Chirugie.*—In one, the teeth were removed from the œsophagus after œsophagotomy; cure in fourteen days. In the second case the foreign body had reached the stomach and was extracted by gastrotomy. In this case, after opening the stomach, Billroth was at first not able to find the foreign body, although the organ was pulled forward and the whole hand introduced within the abdomen. At last the body was discovered at the posterior part of the stomach and removed.—*Medical Chronicle.*

